# Radial artery lumen diameter and intima thickness in patients with abdominal aortic aneurysm

**DOI:** 10.1016/j.jvssci.2022.06.001

**Published:** 2022-08-06

**Authors:** Kristian Shlimon, Marcus Lindenberger, Martin Welander, Frida Dangardt, Niclas Bjarnegård

**Affiliations:** a Department of Health, Medicine and Caring Sciences, Division of Diagnostics and Specialist Medicine, Linköping University, Linköping, Sweden; b Department of Cardiology in Linköping, and Department of Health, Medicine and Caring Sciences, Linköping University, Linköping, Sweden; c Department of Thoracic and Vascular Surgery in Östergötland, and Department of Health, Medicine and Caring Sciences, Linköping University, Linköping, Sweden; d Department of Paediatric Radiology and Clinical Physiology, The Queen Silvia Children's Hospital, Sahlgrenska University Hospital, Gothenburg, Sweden; e Department of Molecular and Clinical Medicine, Institute of Medicine, Sahlgrenska Academy, University of Gothenburg, Gothenburg, Sweden

**Keywords:** Aortic aneurysm, Abdominal, Tunica intima, Ultra-high-frequency ultrasound, Atherosclerosis, Hypertension

## Abstract

**Objective:**

Abdominal aortic aneurysm (AAA) is associated with dilatation of central elastic arteries, while it is uncertain whether peripheral muscular arteries are affected. The aim of this study was to investigate radial artery diastolic lumen diameter (LD), wall thickness, and circumferential wall stress (CWS) in patients with AAA.

**Methods:**

We included 130 men with AAA (mean age, 70.4 ± 3.5 years) and 61 men without AAA (mean age, 70.5 ± 3.2 years) in the study. High-frequency ultrasound examination (50 MHz) was used to measure radial artery diameter, wall thickness, and CWS was calculated.

**Results:**

Men with AAA exhibited smaller radial artery LD (2.34 ± 0.42 mm vs 2.50 ± 0.38 mm; *P* < .01), thicker intima (0.094 ± 0.024 mm vs 0.081 ± 0.018 mm; *P* < .001), similar intima-media (0.28 ± 0.05 vs 0.26 ± 0.05 mm; *P* = NS), and lower CWS (42.9 ± 10.2 kPa vs 48.6 ± 11.4 kPa; *P* < .001), compared with controls. Subgroup analyses including all patients showed smaller LD and thicker intima in patients on statin therapy versus no statin therapy and current/ex-smoking versus never smoking. Individuals with hypertension versus no hypertension also presented with thicker intima, but with no difference in LD.

**Conclusions:**

AAAs demonstrated a smaller LD and thicker intima in the radial artery, in contrast with the theory of a general dilating diathesis of the arteries. Apart from AAA, other factors such as atherosclerosis, smoking habits, and hypertension might also be determinants of radial artery caliber and thickness.

**Clinical Relevance:**

The clinical relevance of this study is the added insight into the pathophysiology of abdominal aortic aneurysm (AAA). Today, the management of AAA is focused on reduction of general cardiovascular risk factors and treatment is based on surgical approaches when the AAA is already manifest. By shedding light on unknown pathophysiological aspects of AAA, it will eventually be possible to develop targeted pharmacological treatments to prevent the formation of AAA, to halt disease progression, and to find early cardiovascular markers of AAA.

**Article Highlights**

• **Type of Research:** Single-center case-control study
• **Key Findings:** High-frequency ultrasound imaging of the radial artery in 130 men with abdominal aortic aneurysm (AAA) and 61 age-matched controls showed smaller lumen diameter (AAA 2.34 mm vs controls 2.50 mm; *P* < .01), and thicker intima layer (AAA 0.094 mm vs controls 0.081 mm; *P* < .001) in men with AAA.
• **Take Home Message:** The hypothesis of a general dilating diathesis in men with AAA could not be supported by the present study, where a smaller radial artery lumen diameter was demonstrated.

The aorta can dilate anywhere along its course, but the highest prevalence of aneurysm formation is seen in the abdominal region. Abdominal aortic aneurysm (AAA) is defined as an abdominal aortic diameter of 30 mm or greater.^1^ In modern studies, AAA has a prevalence of approximately 2% in a 65-year-old Swedish male population.^2^ The pathogenesis is multifactorial and includes chronic inflammation, apoptosis of vascular smooth muscle cells, oxidative stress, and extracellular matrix degradation.^3^, ^4^, ^5^

AAA is generally considered a focal disease, but data suggest that it might be a concealed systemic arterial dilating diathesis that promotes the focal aneurysmal growth.^6^, ^7^, ^8^ Histological studies show similar vessel wall morphology of aneurysms regardless of location.^9^ Previous studies have found wider or less distensible elastic arteries in patients with AAA compared with individuals without AAA.^10^, ^11^, ^12^ However, peripheral muscular arteries are poorly studied, and more studies are needed to explore whether AAA also is associated with peripheral artery remodeling.

Systemic atherosclerosis is common in patients with AAA, but a causal link in aneurysm formation has not been found.^4^ The prevailing view is that atherosclerosis and AAA are two distinct pathological entities that share some common risk factors.^12^^,^^13^ Furthermore, systemic atherosclerosis is positively correlated with increased arterial intima-media thickness and possibly even more strongly with intima thickness (IT).^14^, ^15^, ^16^, ^17^ The proposed general defect in the vasculature in AAA involves the medial layer of arteries whereas coexisting atherosclerosis primarily involves the intimal layer.^6^^,^^9^^,^^18^ It is thus of interest to investigate the thickness of each arterial wall layer in patients with AAA. To our knowledge, no other studies have assessed muscular arteries in the upper extremities with ultra-high-frequency ultrasound examination in individuals with AAA.

The aim of this study was to investigate whether AAA is associated with altered geometry in the distal radial artery. Based on the idea of a general dilating diathesis in AAA, we hypothesize that the radial artery will display a greater diastolic lumen diameter (LD) and increased IT owing to atherosclerosis in patients with AAA.

## Methods

### Patients

Potential study patients were men between 65 and 80 years enrolled in the regional ultrasound surveillance program owing to an AAA diagnosis and men without an AAA diagnosis who had passed an ultrasound screening of the abdominal aorta, all identified from the database at the Department of Clinical Physiology, University Hospital in Linköping, Sweden. Ultrasound screening of the abdominal aorta is offered to all men living in Sweden aged 65 to 70 years. Patients were consecutively contacted by phone, and those who responded and accepted the invitation were offered a prebooked study time. Exclusion criteria were documented irregular heart rhythm, severe cognitive or physical disability, advanced cancer, or language barriers at the time of recruitment.

Finally, 179 men with AAA (aged 70.2 ± 3.8 years), and 75 age-matched controls (70.5 ± 3.2 years) living in the county of Östergötland, Sweden, went through the study protocol. Controls had a maximal infrarenal aortic diameter of 25 mm, and no sign of regional ectasia at their foregoing screening examination. Requirement for AAA diagnosis and inclusion in the present study was an ultrasound-measured maximal diameter of 30 mm within the infrarenal part of aorta, measured according to the leading edge to leading edge principle, at the previous clinical examination.

Information on medical history was retrieved from *International Classification of Diseases*,10th edition, codes in medical records. Ischemic heart disease was defined as having a history of angina, myocardial infarction, and/or chronic ischemic heart disease. Symptomatic cerebrovascular disease was defined as having a history of transient ischemic attack or intracerebral ischemic or hemorrhagic insult.

All patients signed a written informed consent form in accordance with the declaration of Helsinki. The study was approved by the Regional Ethical Review Board at the University of Linköping, Sweden.

### Study protocol

All patients received instructions to refrain from caffeine and tobacco for 4 hours before the study visit. The examinations were performed in a quiet room, where the air temperature was stable between 22 °C and 24 °C. A health questionnaire was used to retrieve information on smoking habits and current medication. After at least 10 minutes of rest in the supine position, upper arm and ankle blood pressures were measured bilaterally. Thereafter, ultrasound scanning of the left radial artery was performed. Finally, blood samples were drawn from a cubital vein and analyzed according to prevailing standardized methods to obtain levels of P-creatinine, P-apolipoprotein A1, P-apolipoprotein B, and B-glycated hemoglobin. The Modification of Diet in Renal Disease equation was used to calculate the estimated glomerular filtration rate.^19^

### Blood pressure measurements and ankle-brachial index

Brachial blood pressure was measured noninvasively with the patient in supine position. The systolic, mean, and diastolic upper arm blood pressures were determined with oscillometric technique (Dinamap PRO 200 Monitor, Critikon, Tampa, FL). The systolic ankle blood pressure was measured by detecting the return of pulsatile blood flow during cuff deflation at the posterior tibial artery and later the dorsalis pedis artery with continuous wave Doppler (Parks model 812, Parks Medical Electronics Inc, Aloha, OR). The ankle-brachial index (ABI) for each leg was calculated by dividing the mean of the posterior tibial artery and dorsalis pedis artery pressure by the higher of the right or left arm systolic blood pressure.^20^

### Vascular ultrasound examination

Ultra-high-frequency ultrasound (Vevo2100, FUJIFILM Visualsonics Inc. Toronto, Canada) equipped with a linear transducer (MS700) was used, with a central frequency of 50 MHz and 30 to 70 MHz bandwidth. The transducer was placed perpendicularly to the axis of the artery in long axis view to clearly visualize the different wall and lumen to wall echo interfaces without applying external compression with the transducer. All radial artery dimensions were determined from a position 1 to 2 cm proximal to the continuous skinfold separating the palm of the hand from the forearm, as has been described in a previous study.^21^ Standard instrument settings were used. Furthermore, only one transmit focus was used and persistence was turned off. The examination was performed by one of three experienced ultrasound operators.

Offline analyses were conducted in Vevo LAB (V 1.7.1, FUJIFILM Visualsonics Inc. Toronto, Ontario, Canada) by a single reader who was blinded to the study groups. The loops were paused in cardiac diastole, defined as the frame when the artery exhibited its smallest diameter, because end-diastole is the standard for reporting the ultrasonic large artery LD.^22^^,^^23^ Two to three digital B-mode cine-loops consisting of one to six consecutive heart beats were saved. Three measurements of radial artery LD, IT, media thickness (MT), and intima-media thickness (IMT) were performed by manual placement of calipers (Fig).

**Fig:**
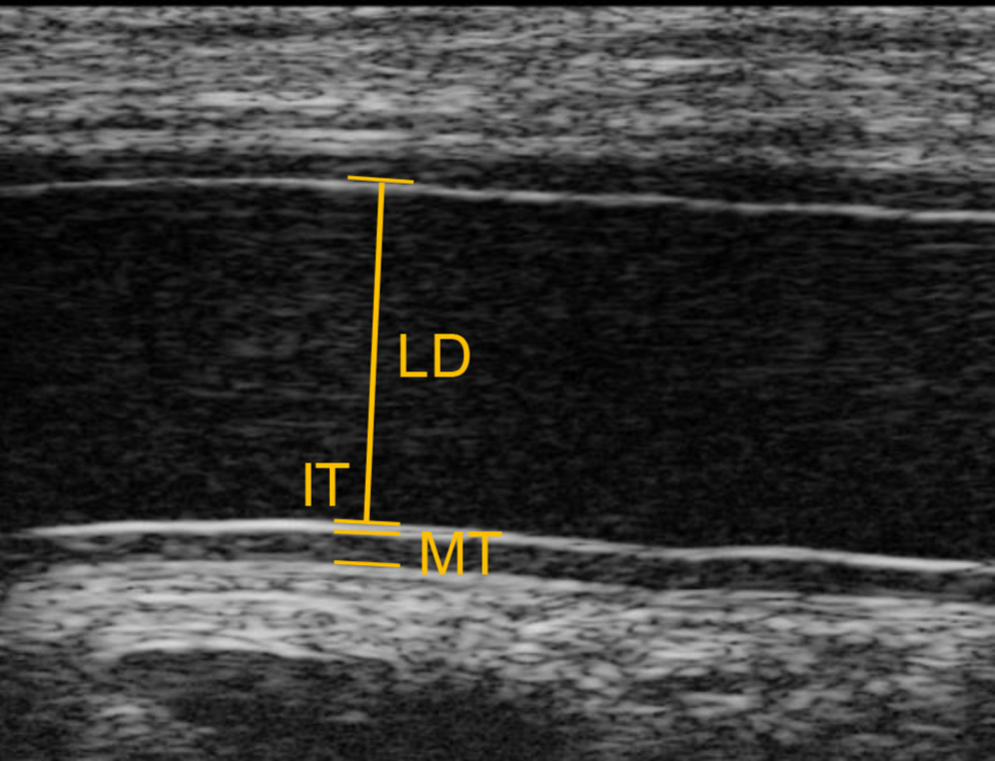
Caliper placement of radial artery LD, far wall IMT and MT. *IMT*, intima-media thickness; *IT*, intima thickness; *LD*, lumen diameter; *MT*, media thickness.

The LD was defined as the distance from the leading edge of the intimal-luminal transition in the near wall to the corresponding edge in the far wall. The IT was defined as the thickness of the first distinct echo (which sometimes may appear as two separate echo lines) of the luminal-intimal transition in the far wall, measured from leading to trailing edge. The MT was defined as the distance from the trailing edge of the IT to the leading edge of the medial-adventitial interface. The leading edge of the medial-adventitial interface was regarded as the first continuous echo in the medial-adventitial interface of the far wall. This measurement has been validated and proposed to represent the trailing border of the MT.^24^ The IMT was defined as the sum of the IT and the MT. All presented data were the mean values taken from two cine-loops.

### Variability

Twenty patients from the present study (15 AAA and 5 controls) were selected randomly for calculations of variability. K.S. analyzed the B-mode images twice, 1 month apart, to assess the intrareader variability. N.B. analyzed the B-mode images on one occasion, masked to study groups and previous results, to assess interreader variability. The intrareader and interreader variability of all the different ultrasound measurements from the radial artery are presented in Supplementary Table I. The variability in the present study is in accordance with previous studies.^18^^,^^21^^,^^25^ In the Supplementary Fig, we also present the test-retest variability (from image acquisition to image analysis) in Bland-Altman graphs with data from a previous internal quality control study.

### Calculations

The radial circumferential wall stress (CWS) is calculated according to the extended form of the law of Laplace.^26^ Total wall thickness is substituted by IMT in the equation, which has been shown to be a feasible approximation in the calculation of wall stress.^27^^,^^28^

Data are presented as kilopascal (kPa); 1 kPa equals approximately 7.50 mm Hg.

$$\text{CWS}\left(\text{kPa}\right)=\frac{\text{PDBP∗LD}}{2\text{∗IMT}}$$

where PDBP is peripheral diastolic blood pressure. The body surface area (BSA) is calculated according to the formula by DuBois and DuBois.^29^

$$\text{BSA}\left({\text{m}}^{2}\right)=0.007184*\text{Height}{\left(\text{cm}\right)}^{0.725}*\text{Weight}{\left(\text{kg}\right)}^{0.425}$$

### Statistical methods

Presented results are expressed as mean ± standard deviation or as percentages. Parametric tests were used for all calculations comparing AAA to controls, relying on the central limit theorem because the studied groups are statistically large enough. The Student's *t* test was used for comparisons between AAA and controls. With the two groups combined (AAA and controls), subgroup analyses of comparisons between patients in regard to hypertension or statin therapy was done with the Student's *t* test, and for smoking habits, analysis of variance was performed. Pearson’s correlation test (r) was used to calculate correlation coefficients. Fisher’s exact test was used for comparisons of disease history and medications between AAA and controls. The intrareader and interreader variabilities were presented with the coefficient of variation and Pearson’s correlation coefficient (r). A two-tailed *P* value of less than .05 was considered statistically significant. SPSS version 27 (IBM Software, Armonk, NY) was used for all statistical analyses.

## Results

### Patient characteristics

During off-line analyses, signs of external radial artery compression or doubtful image quality were found in 60 patients. After exclusion of these 60 patients, all presented data and analysis refers to 130 men with AAA (70.4 ± 3.5 years) and 61 controls (70.5 ± 3.2 years).

Table I presents demographic data, cardiovascular diseases, and smoking habits. No significant differences between AAA and controls were seen in age, body mass index, and height. Brachial blood pressure was similar, and the ABI was lower in AAA than in controls (right leg, 1.12 ± 0.22 vs 1.30 ± 0.17; *P* < .001), albeit in the normal range in both groups. The were 24 (18%) patients with AAA and 2 (3%) in controls with an ABI of less than 0.9 on at least one side (*P <* .01). Current and former smoking status was higher among patients with an AAA (*P <* .001). A history of ischemic heart disease, symptomatic cerebrovascular disease, hyperlipidemia, and hypertension was more prevalent in patients with an AAA than controls: 34 versus 3% (*P <* .01), 13 versus 0% (*P <* .01), 35 versus 20% (*P <* .05), and 74 versus 44% (*P <* .001), respectively.

**Table I:** Clinical characteristics and clinical history of the two cohorts

Clinical characteristics	AAA (n = 130)	Controls (n = 61)	*P* value
Age, years	70.4 ± 3.5	70.5 ± 3.2	NS
Weight, kg	86 ± 13	86 ± 10	NS
Height, m	1.77 ± 0.06	1.78 ± 0.05	NS
BMI, kg/m^2^	27.5 ± 3.8	27.1 ± 2.9	NS
BSA, m^2^	2.03 ± 0.17	2.04 ± 0.13	NS
HR, beats∗min^–1^	60 ± 9	62 ± 10	NS
PSBP, mm Hg	131 ± 19	129 ± 16	NS
PDBP, mm Hg	75 ± 10	74 ± 9	NS
ABI right side	1.12 ± 0.22	1.30 ± 0.17	<.001
ABI left side	1.11 ± 0.22	1.28 ± 0.20	<.001
Clinical history			<.001
Never smoker	17 (13)	33 (54)	
Current smoker	35 (27)	5 (8)	
Ex-smoker	78 (60)	23 (38)	
Hypertension	96 (74)	27 (44)	<.001
Hyperlipidemia	45 (35)	12 (20)	<.05
Type 2 diabetes	15 (12)	5 (8)	NS
Ischemic heart disease	44 (34)	2 (3)	<.01
Cerebrovascular disease	17 (13)	0 (0)	<.01

*AAA*, Abdominal aortic aneurysm; *ABI*, ankle-brachial index; *BMI*, body mass index; *BSA*, body surface area; *HR*, heart rate; *NS*, not significant; *PSBP*, peripheral systolic blood pressure; *PDBP*, peripheral diastolic blood pressure.

Continuous variables are presented as mean ± standard deviation, and categorical variables as number (%).

Table II presents current medication and laboratory data in AAA and controls. The use of antihypertensive drugs and statins were significantly more prevalent in patients with an AAA than in controls: 70 versus 39% (*P <* .001) and 73 versus 20% (*P <* .001), respectively.

**Table II:** Medication, and laboratory data of the two cohorts

Medication	AAA (n = 130)	Controls (n = 61)	*P* value
Antihypertensive therapy	91 (70)	24 (39)	<.001
ACE/ATII inhibitors	68 (52)	17 (28)	<.01
β-blockers	55 (42)	7 (11)	<.001
Calcium channel blockers	35 (27)	9 (15)	NS
Diuretics	26 (20)	6 (10)	NS
Statin therapy	95 (73)	12 (20)	<.001
Laboratory data			
eGFR, mL/min/1.73 m^2^	80 ± 22	86 ± 16	<.05
P-ApoB/ApoA1	0.79 ± 0.25	0.77 ± 0.21	NS
B-HbA1c, mmol/mol^a^	42 ± 12	39 ± 6	NS

*AAA*, Abdominal aortic aneurysm; *ACE/ATII*, angiotensin-converting enzyme/angiotensin II; *ApoA1*, apolipoprotein A1; *ApoB*, apolipoprotein B; *eGFR*, estimated glomerular filtration rate; *HbA1c*, hemoglobin A1c; *NS*, not significant.

Continuous variables are presented as mean ± standard deviation, and categorical variables as number (%).

a Data were available for a smaller population; AAA, n = 96; controls, n = 31.

Patients with cine-loops showing any signs of external arterial compression were excluded from further evaluation. In total, 60 patients (49 AAA and 11 controls) were excluded after file evaluation owing to visible sign of vessel compression or poor image quality. Clinical characteristics, clinical history, and laboratory data for excluded patients are given in Supplementary Tables II and III.

### Radial artery LD

The LD of the radial artery was smaller in patients with an AAA: 2.34 ± 0.42 mm versus 2.50 ± 0.38 mm in controls (*P <* .01; Table III). As seen in Table IV, subgroup analyses across the AAA group and controls presented smaller LDs in individuals on statin therapy compared with individuals not on statin therapy (2.33 ± 0.43 mm vs 2.46 ± 0.37 mm; *P* < .05). A similar difference was seen for current smokers and ex-smokers compared with never smokers (2.33 ± 0.37 mm vs 2.35 ± 0.43 mm vs 2.58 ± 0.37 mm; *P* < .01). A positive correlation was seen between LD and BSA (r = 0.21; *P* < .01; Supplementary Table IV).

**Table III:** Ultrasound measurements from the radial artery

	AAA (n = 130)	Controls (n = 61)	*P* value
LD, mm	2.34 ± 0.42	2.50 ± 0.38	<.01
IT, mm	0.094 ± 0.024	0.081 ± 0.018	<.001
MT, mm	0.18 ± 0.04	0.18 ± 0.04	NS
IMT, mm	0.28 ± 0.05	0.26 ± 0.05	NS
IT/LD	0.042 ± 0.017	0.033 ± 0.010	<.001
MT/LD	0.080 ± 0.022	0.074 ± 0.020	NS
IMT/LD	0.12 ± 0.03	0.11 ± 0.03	<.01
CWS, kPa	42.9 ± 10.2	48.6 ± 11.4	<.001

*AAA*, Abdominal aortic aneurysm; *CWS*, circumferential wall stress; *IMT*, intima-media thickness; *IT*, intima thickness; *LD*, lumen diameter; *MT*, media thickness; *NS*, not significant.

1 kPa = approximately 7.50 mm Hg. Data presented as mean values ± standard deviation.

**Table IV:** Stratification of the radial artery data

		Statin intake		Hypertension		Smoker		
		No (n = 81)	Yes (n = 107)	No (n = 68)	Yes (n = 123)	Never (n = 37)	Ex (n = 101)	Current (n = 40)
LD (mm)								
All		2.45^a^	2.33	2.45	2.36	2.58^b^	2.35	2.33
AAA (n = 130)	2.34^b^	2.40	2.31	2.41	2.31	2.70 (8)	2.32	2.31
Controls (n = 61)	2.50	2.50	2.50 (12)	2.49	2.52 (27)	2.55	2.46 (23)	2.45 (5)
IT (mm)								
All		0.081^c^	0.097	0.082 c	0.094	0.079^b^	0.092	0.094
AAA (n = 130)	0.094^c^	0.084	0.097	0.090	0.095	0.077 (8)	0.094	0.097
Controls (n = 61)	0.081	0.079	0.097 (12)	0.074	0.091 (27)	0.079	0.085 (23)	0.076 (5)
IMT (mm)								
All		0.26^b^	0.28	0.28^b^	0.26	0.25	0.28	0.28
AAA (n = 130)	0.28	0.27	0.28	0.27	0.28	0.27 (8)	0.27	0.28
Controls (n = 61)	0.26	0.25	0.31 (12)	0.24	0.29 (27)	0.25	0.28 (23)	0.27 (5)

*AAA*, Abdominal aortic aneurysm; *HT*, hypertension; *LD*, lumen diameter; *IT*, intima thickness; *IMT*, intima-media thickness.

Data are presented as mean values. Comparison of radial artery data between AAA and controls, stratified for three potential confounders. Numbers in parentheses indicate number of observations when n < 30.

a *P* < .05.

b *P* < .01.

c *P* < .001.

### Radial artery wall thickness and CWS

The intima was thicker in the patients with an AAA compared with controls (0.094 ± 0.024 mm vs 0.080 ± 0.018 mm; *P* < .001) (Table III), although no differences were seen between the groups for MT (AAA 0.18 ± 0.04 mm vs controls 0.18 ± 0.04 mm; *P* = NS) and IMT (AAA 0.28 ± 0.05 mm vs controls 0.26 ± 0.05 mm; *P* = NS). The IT/LD and IMT/LD were significantly higher in AAA vs controls (0.042 ± 0.017 vs 0.033 ± 0.010; *P* < .001) and (0.12 ± 0.03 vs 0.11 ± 0.03; *P* < .01). The CWS was lower in AAA compared with controls (42.9 ± 10.2 kPa vs 48.6 ± 11.4 kPa; *P* < .001).

In subgroup analyses across AAA and controls presented in Table IV, the intima was thicker in individuals on statin therapy versus no statin therapy (0.097 ± 0.025 mm vs 0.081 ± 0.015 mm; *P* < .001); with hypertension versus no hypertension (0.094 ± 0.024 mm vs 0.082 ± 0.017 mm; *P* < .001) and current/ex-smoking versus never smoking (0.094 ± 0.027 mm vs 0.092 ± 0.021 mm vs 0.079 ± 0.015, respectively; *P* < .01).

## Discussion

The main findings of the present study were that (1) patients with an AAA showed smaller radial artery LD and thicker tunica intima compared with age-matched controls and (2) statin therapy as a marker of historical hyperlipidemia, a history of hypertension, and smoking were all related to a smaller radial artery LD and a thicker intimal layer.

### Radial artery LD

Patients with an AAA had a significantly smaller LD compared with controls (Table III). Further data stratification implies that patients on statin therapy, with hypertension, or a smoking history present with smaller LD (Table IV).

It has been proposed that AAA is a general defect in the vasculature with focal manifestation in the abdominal aorta with systemic vascular dilatation, described as the hypothesis of a general dilating diathesis,^7^^,^^10^ at least in elastic arteries, but it remains unclear whether muscular arteries are involved. Our data refute a general dilating diathesis involving the muscular radial artery. This absence of muscular artery dilatation is in accordance with our previous observations on muscular arteries in the lower limbs, where similar diameter of the common femoral artery and popliteal artery were found in patients with AAA and healthy patients.^30^ This finding contrasts with a study by Ward,^6^ who found wider brachial, popliteal, and common femoral arteries in patients with AAA in comparison with controls. In the study by Ward,^6^ patients with AAA were outpatients, whereas controls were recruited from a cohort awaiting vascular or urological surgery. This strategy is in distinction to the present study, where controls were recruited from a regional screening program, which better represents a general population. It has also been shown that upper limb venous compliance and capacitance are decreased in patients with AAA,^31^ further supporting the view that AAA might affect vascular physiology and geometry distant from the aorta.

We believe that the observed differences in the radial artery LD between patients with AAA and controls to some extent reflects differences in overall cardiovascular disease rather than AAA per se, because data stratification for statin therapy, hypertension, and smoking also showed a smaller LD. Similar observations have been shown in a recent review article, where cardiovascular comorbidity is associated with smaller radial artery LD.^32^ Hypertension has not previously been shown to affect LD of the radial artery,^32^^,^^33^ which is in line with our results (Table IV).

### Radial artery wall thickness and CWS

AAA exhibited both thicker intima and increased intima-lumen-ratio compared with controls (Table III). Statin therapy, hypertension, and/or smoking were also associated with a higher IT (Table IV). Histologically, AAA seems to mainly affect the medial layer of the abdominal aorta.^9^ However, in the present study the radial artery MT (Table III) was similar across AAA and controls, further contradicting that the hypothesized systemic vascular pathogenesis leading to AAA would affect muscular arteries.

AAA and atherosclerosis often coexist, but affect the arterial intima and media differently.^3^ The IT of the radial artery has not been studied in patients with AAA earlier, but could be increased on the basis of atherosclerosis as shown in the radial artery and common carotid artery in patients with cardiovascular disease, further illustrating the potential advantage of examining IT separately.^17^^,^^34^ Higher radial artery IT and IMT have been shown to be associated with higher risk of future cardiovascular events.^18^^,^^35^ Moreover, serum levels of P-apolipoprotein B and P-apolipoprotein A1 have been shown to greatly correlate with the presence of vascular atherogenesis.^36^ It has also been shown that end-stage renal disease may increase the IT of the radial artery.^37^ In the present study, however, the estimated glomerular filtration rate was clinically similar between the groups.

The higher statin use in the AAA cohort might have lowered their apolipoprotein ratio and masked a true historical difference between the groups, altogether making existent apolipoprotein data hard to interpret. Patients on statin therapy exhibited a thicker intima as a sign of prolonged duration of hyperlipidemia versus patients on no statin therapy (Table IV), despite some studies indicating that statin treatment over time may halt IMT progression rate in patients with cardiovascular disease.^38^^,^^39^

In mice models, hypertension may induce intimal hyperplasia through damage of vascular endothelium and inflammation.^40^^,^^41^ In our study, the radial artery intima was indeed thicker in individuals with hypertension (Table IV), in accordance with other studies measuring radial artery IT in individuals with hypertension.^42^ The radial artery has in earlier studies been shown to mechanically fully adapt to hypertensive settings leading to increased IMT, preserved LD, and unchanged CWS in patients with hypertension compared with nonhypertensive patients.^43^^,^^44^ In patients with AAA, CWS was lower in the present study, further indicating that other factors than hypertension affecting the radial artery are involved, such as atherosclerosis.

The role of smoking in carotid artery intima-media thickening and narrowing of LD is relatively established,^45^^,^^46^ in contrast with the radial artery. In our study, smoking was more prevalent in the AAA group, as expected when considering the strong association between AAA and smoking.^5^ Interestingly, the intima layer, but not the full intima-media complex, was thicker in smokers (Table IV). This finding might imply that the IT in the radial artery is a more sensitive marker of arterial injury than the IMT.

### Limitations

The superficial course of the distal radial artery makes it prone to external compression. To secure the validity of the B-mode distance measurements, it was necessary to exclude patients who showed signs of external compression from the study. In addition, information on radial artery accesses (puncture, cannulation, etc) before the study is missing.

The number of potential controls in the screening program is far greater than potential patients with AAA, which comes with the risk of skewed selection. Recruitment of patients was performed consecutively through phone calls during office hours. Additionally, the regional AAA screening program is implemented in the whole county, but to restrict long travel distances to the study center, only males living in the municipality of Linköping were invited, which may have introduced a selection bias.

Finally, we made no statistical adjustment for potential confounders when analyzing the radial artery parameters because many of these are interlinked and risk factors of, or in some way associated with AAA, such as, statin therapy, hypertension, and smoking. Instead, we performed subgroup analyses stratified by the main potential confounders of interest, namely, to investigate alternative explanations for group differences. However, the introduction of subgroups also changes the distribution of other potential confounders, and this limitation should be noted when interpreting the results. Propensity score stratification has earlier been proposed to decrease the effect of confounding in observational studies, but it was not possible to perform in this study because there were too few patients in some propensity score strata.^47^ The same problem applied to the subgroup analyses, which prevented us from statistically investigating differences between AAA and controls stratified by potential confounders.

## Conclusions

Altered radial artery geometry was found in patients with AAA, presenting with a smaller lumen diameter, a thicker intima layer, and a concomitantly lower circumferential wall stress. The theory of a general dilatation in AAA cannot be supported by findings in the present study.

## Author Contributions

Conception and design: NB

Analysis and interpretation: KS, ML, NB

Data collection: KS, MW, NB

Writing the article: KS

Critical revision of the article: KS, ML, MW, FD, NB

Final approval of the article: KS, ML, MW, FD, NB

Statistical analysis: KS, NB

Obtained funding: ML

Overall responsibility: NB

## Figures and Tables

**Supplementary Fig:**
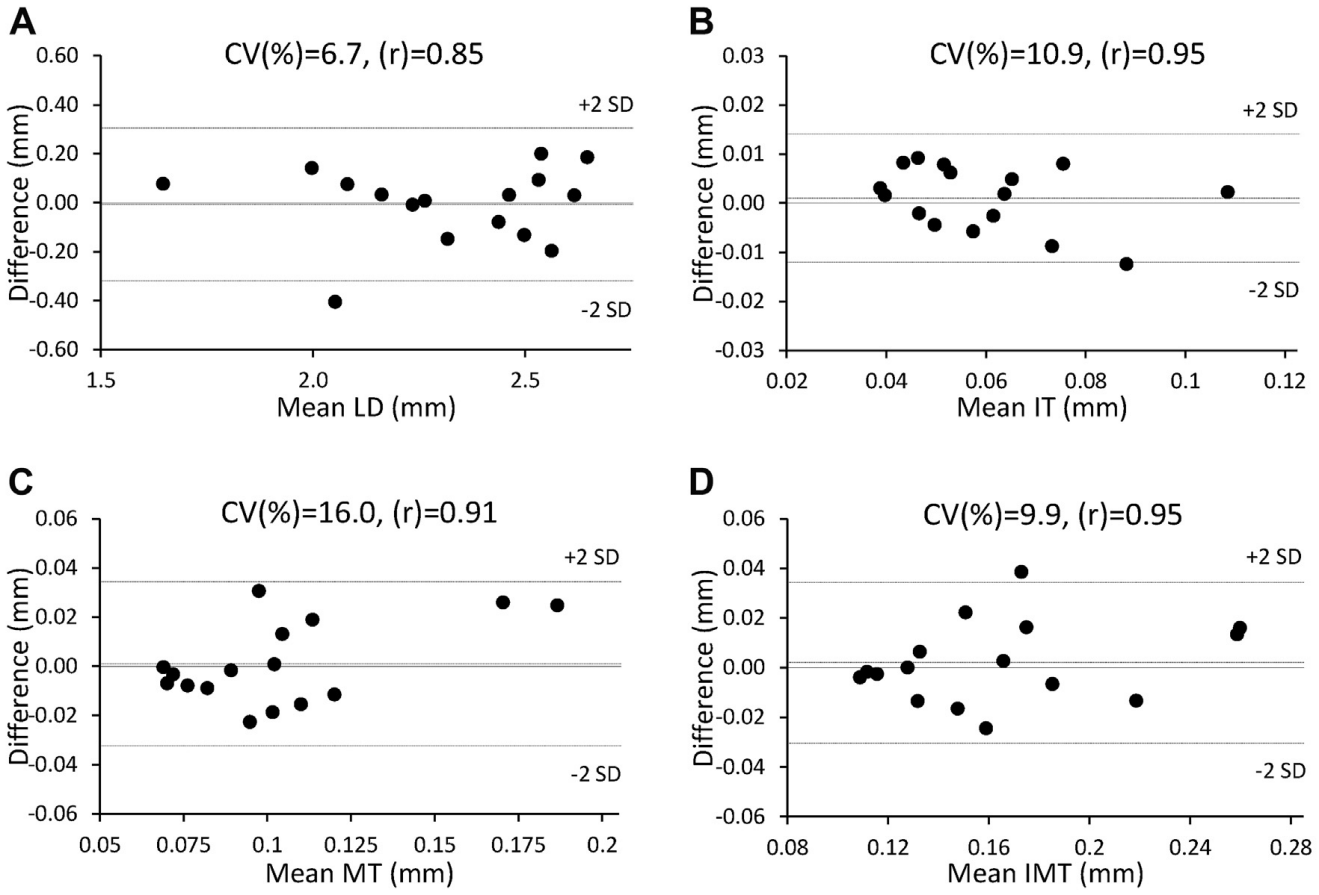
Test-retest variability of radial artery B-mode ultrasound measurements. Bland-Altman plot of the test-retest variability (from image acquisition to image analysis) of lumen diameter (LD) **(A)**, intima thickness (IT) **(B),** media thickness (MT) **(C)**, and intima-media thickness (IMT) **(D)**. Lines represent zero difference and mean average ± 2 standard deviations. *CV*, coefficient of variation; *r*, Pearson correlation coefficient. All compiled data are from an earlier methodological study where healthy volunteers between 16 and 85 years were reexamined 2 weeks after their first visit by a single ultrasound technician (N.B.). Later, a single reader (K.S.) determined radial artery parameters on all the saved B-mode images in a blinded manner. The purpose was to explore the total variability, taking the methodological as well as the biological variability into account.

**Supplementary Table I:** Measurement variability of ultrasound images

	Intra-reader variability		Inter-reader variability	
	CV (%)	(r)	CV (%)	(r)
LD	1.2	1.00	3.5	0.97
IT	9.0	0.93	11.9	0.88
MT	4.4	0.99	11.1	0.92
IMT	4.4	0.98	7.2	0.96

*CV*, Coefficient of variation; *IMT*, intima-media thickness; *IT*, intima thickness; *LD*, lumen diameter; *MT*, media thickness; *r*, Pearson correlation coefficient.

Manual distance measurements on ultrasound files from 20 randomly selected patients in the present study were performed on two occasions to assess the reader variability.

**Supplementary Table II:** Clinical characteristics of excluded patients

Clinical characteristics	AAA (n = 49)	Controls (n = 11)
Age, years	69.8 ± 4.5	69.1 ± 1,9
Weight, kg	88 ± 14	86 ± 15
Height, m	1.77 ± 0.06	1.77 ± 0.06
BMI, kg/m^2^	28.2 ± 4.3	27.6 ± 4.0
BSA, m^2^	2.05 ± 0.15	2.03 ± 0.18
HR, min^–1^	60 ± 8	56 ± 8
PSBP, mm Hg	132 ± 18	128 ± 13
PDBP, mm Hg	75 ± 7	73 ± 6
ABI right side	1.17 ± 0.18	1.22 ± 0.04
ABI left side	1.16 ± 0.17	1.22 ± 0.09
Clinical history		
Never smoker	4 (8)	7 (70)
Current smoker	16 (33)	0 (0)
Ex-smoker	29 (59)	3 (30)
Hypertension	42 (85)	2 (18)
Hyperlipidemia	33 (67)	1 (9)
Type 2 diabetes	7 (14)	4 (36)
Ischemic heart disease	23 (47)	1 (9)
Cerebrovascular disease	5 (10)	0 (0)

*AAA*, Abdominal aortic aneurysm; *ABI*, ankle-brachial index; *BMI*, body mass index; *BSA*, body surface area; *HR*, heart rate; *PDBP*, peripheral diastolic blood pressure; *PSBP*, peripheral systolic blood pressure.

Continuous variables are presented as mean ± standard deviation, and categorical variables as number (%).

**Supplementary Table III:** Medication, and laboratory data of excluded patients

Medication	AAA (n = 49)	Controls (n = 11)
Antihypertensive therapy	39 (80)	3 (27)
ACE/ATII inhibitors	26 (53)	2 (18)
β-Blockers	20 (41)	3 (27)
Calcium channel blockers	20 (41)	0 (0)
Diuretics	9 (18)	1 (9)
Statin therapy	37 (76)	2 (18)
Laboratory data		
eGFR, mL/min/1.73 m^2^	85 ± 20	81 ± 18
P-ApoB/ApoA1	0.84 ± 0.28	0.76 ± 0.22
B-HbA1c, mmol/mol	41 ± 6	46 ± 14

*AAA*, Abdominal aortic aneurysm; *ACE/ATII*, angiotensin-converting enzyme/angiotensin II; *ApoA1*, apolipoprotein A1; *ApoB*, apolipoprotein B; *eGFR*, estimated glomerular filtration rate; *HbA1c*, hemoglobin A1c.

Continuous variables are presented as mean ± standard deviation, and categorical variables as number (%).

**Supplementary Table IV:** Correlation coefficients between vessel parameters and patient characteristics

Correlations (Pearsons's r)	LD	IT	IMT
Age	–0.06	0.02	–0.01
BMI	0.10	0.02	0.25^a^
BSA	0.21^a^	–0.05	0.16^b^
eGFR	–0.08	–0.02	–0.01
IHD	–0.19^a^	0.17^b^	0.06
HT	–0.11	0.26^a^	0.20^a^
Statin therapy	–0.15	0.36^a^	0.23^a^

*BMI*, Body mass index; *BSA*, body surface area; *eGFR*, estimated glomerular filtration rate; *HT*, hypertension; *IHD*, ischemic heart disease; *IMT*, intima-media thickness; *IT*, intima thickness; *LD*, lumen diameter.

a *P* < .01.

b *P* < .05.
